# The putative C-type lectin Schlaff ensures epidermal barrier compactness in *Drosophila*

**DOI:** 10.1038/s41598-019-41734-9

**Published:** 2019-03-29

**Authors:** Renata Zuber, Khaleelulla Saheb Shaik, Frauke Meyer, Hsin-Nin Ho, Anna Speidel, Nicole Gehring, Slawomir Bartoszewski, Heinz Schwarz, Bernard Moussian

**Affiliations:** 1 Applied Zoology, Technical University of Dresden, Zellescher Weg 20b, 01217 Dresden, Germany; 2 University of Tübingen, Interfaculty Institute of Cell Biology, Section Animal Genetics, Auf der Morgenstelle 15, 72076 Tübingen, Germany; 3 Rzeszow University, Department of Biochemistry and Cell Biology, ul. Zelwerowicza 4, 35-601 Rzeszów, Poland; 4 Max-Planck-Institut für Entwicklungsbiologie, Microscopy Unit, Spemannstr. 35, 72076 Tübingen, Germany; 5 Université Côte d’Azur, CNRS, Inserm, Institute of Biology Valrose, Parc Valrose, 06108 Nice CEDEX 2, France

## Abstract

The stability of extracellular matrices is in general ensured by cross-linking of its components. Previously, we had shown that the integrity of the layered *Drosophila* cuticle relies on the presence of a covalent cuticular dityrosine network. Production and composition of this structure remained unstudied. In this work, we present our analyses of the *schlaff* (*slf*) gene coding for a putative C-type lectin that is needed for the adhesion between the horizontal cuticle layers. The Slf protein mainly localizes between the two layers called epicuticle and procuticle that separate from each other when the function of Slf is reduced or eliminated paralleling the phenotype of a cuticle with reduced extracellular dityrosine. Localisation of the dityrosinylated protein Resilin to the epicuticle-procuticle interface suggests that the dityrosine network mediates the adhesion of the epicuticle to the procuticle. Ultimately, compromised Slf function is associated with massive water loss. In summary, we propose that Slf is implied in the stabilisation of a dityrosine layer especially between the epicuticle and the procuticle that in turn constitutes an outward barrier against uncontrolled water flow.

## Introduction

Extracellular matrices (ECM) contribute to tissue shape and function. Their integrity depends on covalent and non-covalent interaction of their components. Collagen crosslinking in the articular cartilage by lysyl oxidases, for example, enhances tissue stability against physical wears^1^. Another prominent example is the apical extracellular layered network of lipids and proteins that constitutes the epidermal stratum corneum^2–4^. A defective stratum corneum in patients suffering different types of ichthyoses provokes a dry and scaly skin^5^. Lamellar ichthyosis is caused by mutations in the gene encoding the central cross-linking enzyme transglutaminase that introduces covalent glutamine-lysine bonds. Extracellular dityrosine links catalysed by peroxidases have been identified in connective tissues and in response to oxidative stress^6–11^. While the molecular mechanisms and biochemical reactions of ECM network formation are well understood, the subcellular localisation of these processes are largely unexplored.

We address this issue by studying the molecular and cellular processes of insect cuticle differentiation. The insect cuticle is an ECM that consists of the polysaccharide chitin, proteins, catecholamines and lipids that interact with each other to form a layered structure including the outermost envelope, the middle epicuticle and the inner procuticle^12–15^. It is produced and organised at the apical plasma membrane and in the region adjacent to it named the assembly zone. In the fruit fly *Drosophila melanogaster*, several proteins including Knickkopf (Knk), Obstractor A (Obst-A), Chitinase 2 and the chitin deacetylases Vermiform (Verm) and Serpentine (Serp) act in concert to ensure the stereotypic organisation of the larval cuticle during embryogenesis and larval development^16–19^. Stabilisation of the cuticle depends partly on a network of molecular bonds between different types of yet largely unknown proteins mediated by catecholamines, glutamine-lysine bridges and dityrosines^20–22^. Catecholamine incorporation depends on a set of insect-specific enzymes including extracellular phenol-oxidases (sclerotisation) and occurs predominantly within the upper region of the procuticle called exocuticle while its lower regions, named endocuticle, remains unsclerotised^23,24^. In the red flour beetle *Tribolium castaneum*, for instance, the phenol-oxidase Laccase2 catalyses the cross-link between the cuticle proteins TcCP30, TcCpr18 and TcCpr27 thereby stabilising the cuticle matrix and shape^25^. Glutamine-lysine crosslinking in *D. melanogaster* involves a transglutaminase that among others uses the chitin-binding proteins Cpr76Bd, Cpr47Ef, Cpr64Ac and Cpr97Eb as substrates suggesting that it acts within the procuticle. Dityrosine crosslinking was postulated to occur in the basal site of the procuticle adjacent to the apical plasma membrane of the epidermal cells^22^. Spatial information is, hence, available for these events. By contrast, the molecular and cellular mechanisms that control or govern their localisation within the differentiating cuticle are unknown.

In this work, we have analysed the role of the C-type lectin Schlaff (Slf) in cuticle organisation and compactness in *D. melanogaster* in a genetic approach. We demonstrate that Slf participates in the establishment of the dityrosine network within a distinct zone of the cuticle required for overall stability of the ECM.

## Results

### Cuticle phenotype of slf mutant larvae

**Figure 1:**
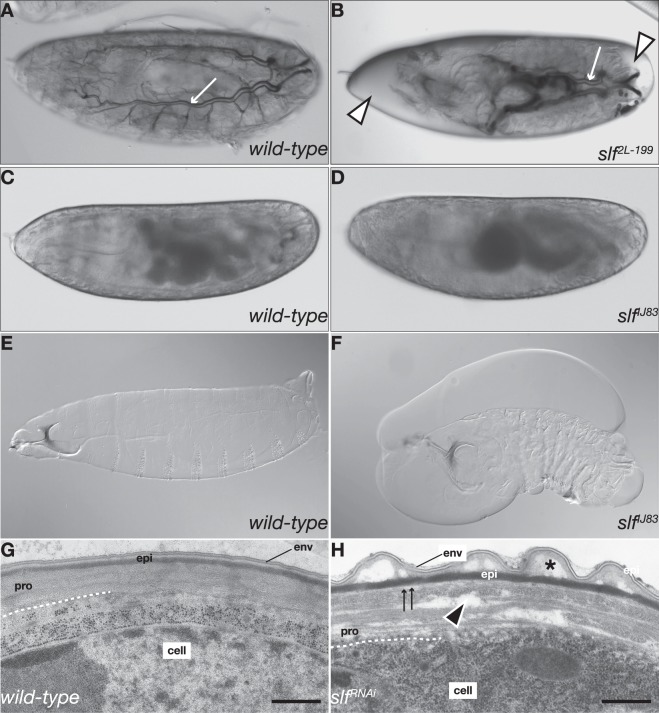
Homozygous *slf* mutant larvae contract and lose water whilst their cuticle detaches from the body surface. The ready-to-hatch living *wild type* larva fills the entire egg space (**A**), whilst the homozygous *slf* mutant larva (**B**) is separated from the egg case by liquid (white triangles). The head skeleton and the tracheae (white arrow) are unaffected in these larvae. Before tracheal air-filling, at mid-stage 17, the wild-type (**C**) and the *slf* ^*IJ83*^ mutant embryos (**D**) both occupy the entire egg space. The cuticle of the wild type larvae fixed in Hoyer’s medium acquires a spindle-like shape (**E**) whilst the cuticle of *slf* mutant larvae forms irregular bulges, especially in the head region, whereas the abdominal cuticle is crumpled (**F**). The cuticle of ready-to-hatch *wild type* larvae in transmission electron micrographs (**G**) is built of three distinct and tightly adhering layers: the external envelope (env) consisting of alternating electron-lucid and electron-dense sheets, the bipartite epicuticle and the chitinous procuticle (pro) that consists of chitin sheets (laminae). In *slf* mutant larvae (**H**) the procuticular laminar organization is disrupted by various sizes of electron-lucid regions (black triangle). Adhesion between the epicuticle and the procuticle is disrupted (arrows). The structure of the envelope seems to be normal but the envelope may detach from the epicuticle (asterisk).

Differentiation of the *D. melanogaster* larval cuticle is initiated at stage 15 of embryogenesis and ends shortly before hatching^26^. The developing embryo and the ready-to-hatch larva almost fills the entire space of the egg (Fig. 1). Homozygous *slf* mutant embryos look normal when cuticle differentiation starts, but ready-to-hatch larvae retract from the egg-shell and the space between the larva and the egg-shell is filled with liquid (Fig. 1). When freed from the egg, the larvae contract and crumple and the cuticle occasionally detaches from the surface of the animal (Supplementary Fig. S1). The cuticular structures head skeleton and tracheae are, however, unaffected. When fixed with Hoyer’s medium, the cuticle detaches from the body surface and forms blisters (Fig. 1). Larvae transheterozygous for an EMS-induced *slf* mutation and any deficiency uncovering the *slf* locus e.g. Df(2 L)ED250 or Df(2 L)BSC225 (see below) display the same phenotype as *slf* homozygous mutant larvae. Thus, Slf is essential for epidermal cuticle integrity, but dispensable for the intactness of the head skeleton and the tracheal cuticle. Overall, the *slf* mutant phenotype is reminiscent of the phenotype caused by a deletion of the *alas* gene that codes for an enzyme of the heme biosynthesis pathway^22^.

**Figure 2:**
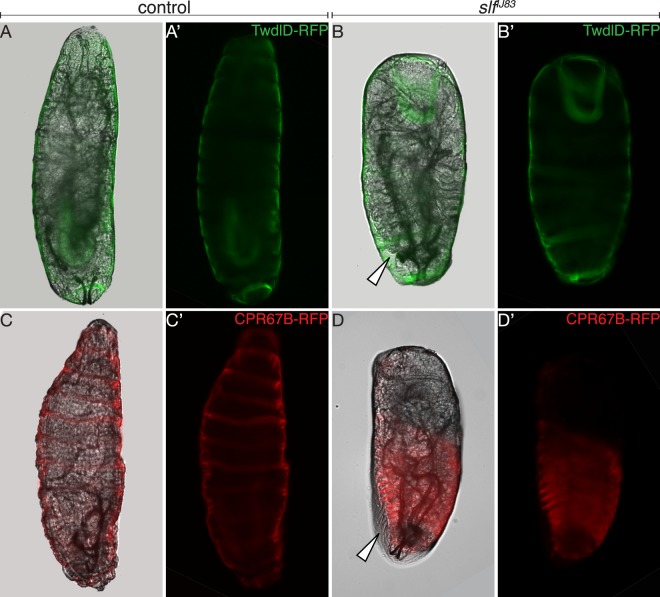
Cuticle of *slf* mutant larvae is delaminated. The layer of TweedleD (TwdlD-dsRed, green) lines the body in living *wild type* larvae before hatching mounted in hydrocarbon medium (**A,A’**), whilst in *slf* homozygous mutant larvae this layer detaches from the body surface (**B,B’**, white arrow). The layer represented by CPR67b-RFP lines the body in *wild type* (**C,C’**) and *slf* (**D,D’**) mutant larvae, in spite of the detachment of the other cuticular layers in *slf* mutant larvae (white arrow).

For a detailed analysis of the cuticle phenotype, we examined the localisation of fluorescent-tagged cuticular proteins in wild-type and *slf* mutant larvae (Fig. 2 and Supplementary Fig. S2). We chose two predicted cuticle proteins that according to flybase information (flybase.org) are expressed during late embryogenesis and in the first instar larvae: TweedleD-dsRed (TwdlD-dsRed)^27^ with an unknown subcellular localisation and the putative chitin-binding Cuticular Protein 67B-RFP (CPR67B-RFP) with a probable localisation to the procuticle. Both chimeric proteins line the body surface of the wild-type larva. In *slf* mutant larvae, the region marked by TwdlD-dsRed detaches from the epidermis. By contrast, the region of CPR67B-RFP localisation does not detach and marks the body surface of these larvae (Fig. 2).

Taken together, mutations in *slf* affect the integrity of the larval body. Especially, the adhesion between the TwdlD-dsRed and CPR67B-RFP domains within the epidermal cuticle depends on Slf. We assume that the observed liquid in the egg space of *slf* mutant embryos is the haemolymph that leaks out through the defective cuticle.

### Slf is not needed for the function of the inward barrier

To further inspect cuticle barrier function, we performed a dye penetration assay that we had developed recently^28,29^. Incubation of wild-type, *slf* and *alas* mutant ready-to-hatch embryos with bromophenol blue does not result in dye uptake, while *snurstorr snarlik* (*snsl*) mutant animals with a defective envelope^30^ do so (Supplementary Fig. S3). Thus, Slf is not needed for protection of xenobiotic penetration through the cuticle.

### The cuticle of slf mutant embryos is delaminated

In order to understand the defects at the cellular level, we analysed the ultrastructure of the body cuticle of *slf* mutant larvae by transmission electron microscopy (Fig. 1). The wild-type body cuticle is composed of three biochemically distinct horizontal layers, the envelope, the epicuticle and the procuticle. The upper envelope consists of alternating electron-dense and electron-lucid films. The middle epicuticle is a bipartite matrix of cross-linked proteins and lipids. The procuticle contacting the apical surface of the epidermal cell is characterised by a helicoidal stack of chitin-protein sheets (laminae). In the cuticle of *slf* mutant ready-to-hatch larvae unstructured regions of various sizes disrupt the organisation of the laminae. The procuticle is occasionally separated from the above epicuticle. The upper tier of the epicuticle is not smooth. The envelope is continuous and its ultrastructure appears to be normal. In summary, in *slf* mutant larvae, cuticle compactness along the vertical axis is lost.

### Mutations in slf do not affect septate junctions

Loss of epithelial barrier function is observed in *Drosophila* embryos that have mutations in genes coding for septate junction (SJ) components^31^. To test whether *slf* mutations affect SJ integrity in the epidermis, we investigated the ultrastructure of the SJ in *slf* mutant embryos by transmission electron microscopy (Supplementary Fig. S4). In the wild-type larva, SJs connect neighbouring epidermal cells. In the *slf* mutant larva the SJ ultrastructure is unchanged. The correct assembly of SJs does not exclude that they may have nevertheless lost their paracellular barrier function. We performed dye injection assays to analyse SJ barrier function (Supplementary Fig. S4). When wild-type stage 16 embryos were injected with 10 kDa dye-conjugated dextran, the epidermal cells retained dextran within the body cavity. In *slf* mutant stage 16 embryos dextran retention is normal. Hence, we conclude that the loss of barrier function in *slf* mutant larvae is not due to defective SJ.

### Slf is a C-type lectin expressed in the epidermis

**Figure 3:**
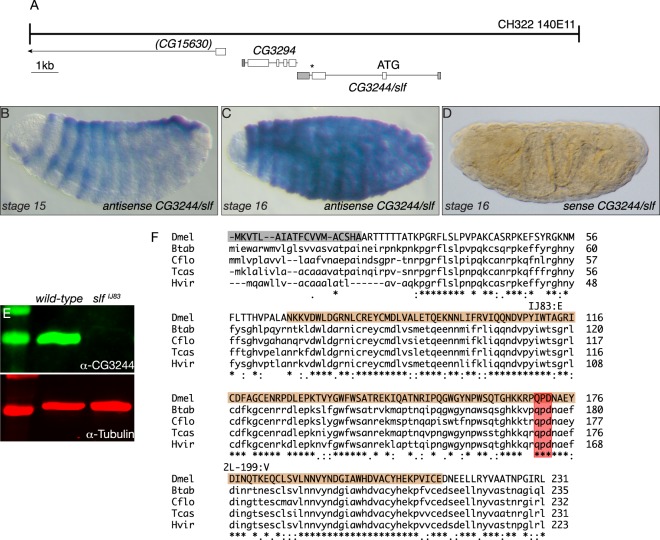
Slf is a putative C-type lectin. The CHS322-140E11 insertion including the complete sequences of the two genes *GC3294* and *CG3244* (ATG and stop codon * indicated) and the partial (brackets) sequence of *CG15630* (line with arrowhead pointing to 3′ region of this gene outside of CHS322-140E11) recombined on the chromosome carrying the *slf* ^*2L–199*^ mutation rescues the *slf* mutant phenotype (**A**). Detection of the *CG3244* transcript in the developing embryos using a *CG3244* antisense probe shows a signal in the epidermis at stage 15 and 16 (**B,C**, blue). The sense probe does not yield any signal in the embryo after hybridization (**D**). In Western blot experiments, antibodies in an anti-serum raised against the Slf protein recognise a band at round 27 kDa matching the predicted 26.3 kDa (flybase) in protein extracts of wild-type embryos (**E**). This band is missing in extracts from *slf* ^*IJ83*^ embryos suggesting that the respective protein is unstable. The Slf protein is composed of the N-terminal signal peptide (light grey box) and the C-type lectin domain (CTLD, cd00037, brown box) with the QPD motif (red box), found in galactose binding lectins (**F**). Putative orthologues are found in major insect orders represented for example by *Tribolium castaneum* (Tcas, Coleoptera, LOC657853, 87% similarity), *Camponotus floridanus* (Cflo, Hymenoptera, LOC105258283, 87% similarity), *Heliothis virescens* (Hvir, Lepidoptera, B5V51_5061, 89% similarity) and *Bemisia tabaci* (Btab, Hemiptera, LOC109037209, 88% similarity). After sequencing of the *CG3244* gene, in the respective protein sequence of *slf* ^*IJ83*^ mutant embryos an exchange of glycine^114^ (GG^449^A) to glutamate (GAA) was identified, and in the respective protein sequence of *slf* ^*2L–199*^ mutant embryos an exchange of glutamate^183^ (GA^656^G) to valine (GTG) was found. Of note, we did not find any missense or nonsense mutation in the *CG3294* locus in the genomic DNA of *slf* ^*IJ83*^. In the genomic DNA of *slf* ^*2L–199*^ animals, there was one missense mutation changing the penultimate amino acid R^455^ to C without affecting any known domain. This exchange is unlikely to be important as the CG3294 orthologues in *Drosophila erecta* and *Drosophila sedulia*, for instance, have also a C at this position.

In order to understand the molecular defects caused by mutations in *slf*, we identified the gene affected by these mutations. The *slf* gene was initially mapped to the cytological position 25 A to 25 C on the left arm of chromosome 2 (see flybase.org). By deficiency mapping, we localised the mutations in an interval uncovered by Df(2 L)BSC225 containing 10 loci. To narrow down the *slf* region, we attempted to reduce the number of candidate genes in a transgenic rescue experiment. Due to the cuticle defects observed in *slf* mutant larvae, we suspected that the factor affected might be associated with the apical plasma membrane or extracellular. A good candidate is CG3244 (Clect27) that was reported to be needed for wing cuticle integrity^21^. We recombined an insertion of the Pacman CH322-140E11 (20233 bp) that includes *CG3244* and the neighbouring gene *CG3294*, coding for a putative zinc-finger RNA-binding protein associated with wound healing^32^, to the chromosome harbouring the *slf* ^*2L–199*^ mutation (Fig. 3). According to the Fly-FISH database (http://fly-fish.ccbr.utoronto.ca/gene/3770/), *CG3294* is not expressed in the epidermis, whereas according to the FlyExpress database (http://www.flyexpress.net/search/genes/Clect27/images/BDGP/LDVO) *CG3244* is expressed in this tissue. Homozygous *slf* ^*2L–199*^ larvae carrying the CH322-140E11 insertion do not display the *slf* mutant phenotype. In *in situ* experiments, we detected the *CG3244* transcript in the developing epidermis during late embryogenesis when the cuticle is formed (Fig. 3), but not in the head skeleton or the tracheal system.

As predicted by the SignalP software, CG3244 possesses a signal peptide suggesting that it may be secreted. CG3244, a Ca^2+^-dependent lectin (C-type lectin), has recently been proposed to be a target of the transglutaminase that catalyses the cross-linking of proteins in the cuticle^21^. We sequenced the genomic DNA of the candidate *CG3244* isolated from the two EMS alleles *slf* ^*IJ83*^ and *slf* ^*2L–199*^ and identified in each sequence a single point mutation that leads to an exchange of an amino acid (Fig. 3). These amino acids are conserved between CG3244 and homologous sequences of species representing hemi- and holometabolous insects. A polyclonal rabbit antiserum produced against CG3244 failed to recognise an antigen in protein extracts from *slf* ^*IJ83*^ mutant larvae, while a 27 kDa protein was present in protein extracts from wild-type ready-to-hatch embryos (Fig. 3 and Supplementary Fig. S5). Moreover, we were able to phenocopy the *slf*-mutant phenotype by RNA interference (RNAi) through the expression of UAS-driven *CG3244* RNA hairpin constructs in the epidermis (Supplementary Fig. S1). Thus, we conclude that CG3244 represents Slf and that the mutations in *CG3244* are responsible for the *slf* mutant phenotype described above.

The Slf protein contains 231 amino acids and is composed of an N-terminal signal peptide and a C-type lectin domain (Fig. 3). The motif QPD especially within the C-type lectin domain is found in galactose binding lectins^33^. In the fruit fly genome, there are two additional genes coding for Slf homologous proteins, CG4115 and CG6055 (Supplementary Fig. S6). Closely related sequences, probably Slf orthologs are found in other arthropods (Fig. 3). *In silico* searches in protein structure databases revealed a possible structural homology to L-Selectins from vertebrates (Supplementary Fig. 6), which actually do not seem to have true counterparts in *Drosophila*. Taking all these data together, we conclude that *slf* encodes the C-type lectin CG3244, which potentially binds extracellular sugars, probably galactose.

### Slf defines a new zone within the epidermal cuticle

**Figure 4:**
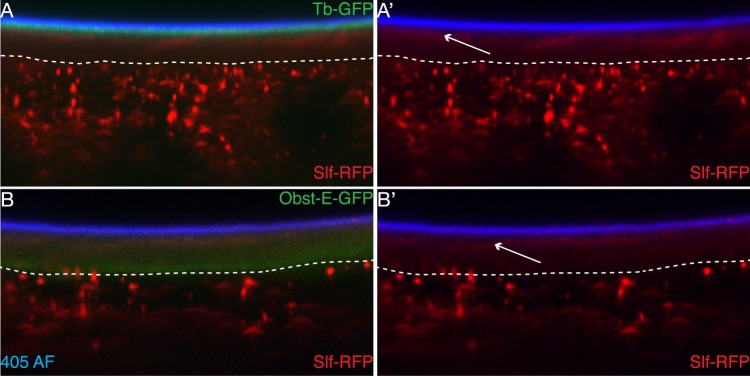
Slf protein localizes between the Tweedle layer and the procuticle. RFP-tagged Slf protein expressed in the epidermis under the control of the promoter of the *tweedleM* gene (*twdlm* > *slf-RFP*) forms particles in the cell and a thin layer in the cuticle of the living third instar larvae (**A,B**). The Slf layer (red) occurs below the Tweedle layer marked by a GFP-tagged Tubby protein, Tb-GFP (green; **A,A’**) and at the upper edge of the chitinous procuticle marked by GFP-tagged ObstE (green; **B,B’**). The 405-nm induced auto-fluorescence (405 AF) of the outermost cuticular layer envelope is marked in blue (**A–B’**).

Loss of cuticle compactness as shown in Fig. 1 suggests that Slf is a coupling link between cuticle components. In order to examine the cuticular localisation of Slf we generated a C-terminally RFP-tagged Slf (Slf-RFP) version expressed in the larval epidermis under the control of the *tweedleM* promoter. To visualize the cuticle, we used a GFP-tagged version of the Tweedle-class protein Tubby (Tb-GFP) and an E-GFP-tagged version of the chitin-binding protein Obstructor (ObstE-GFP)^34^. Tb-GFP marks an apical region, while ObstE-GFP localises to a basal region adjacent to the epidermis. A Slf-RFP signal is detected in the whole procuticle with a strong signal in a thin region just below the Tb-GFP area and at the apical border of the ObstE-GFP layer (Fig. 4). Dots of an RFP signal occurred also under the procuticle, probably depicting intracellular vesicles. These data indicate that Slf localisation within the procuticle is necessary for the adhesion between chitin laminae and between the procuticle and the epicuticle. We speculate that its accumulation in the apical region of the procuticle may define a new cuticle zone.

### Slf is required for soft cuticle integrity

**Figure 5:**
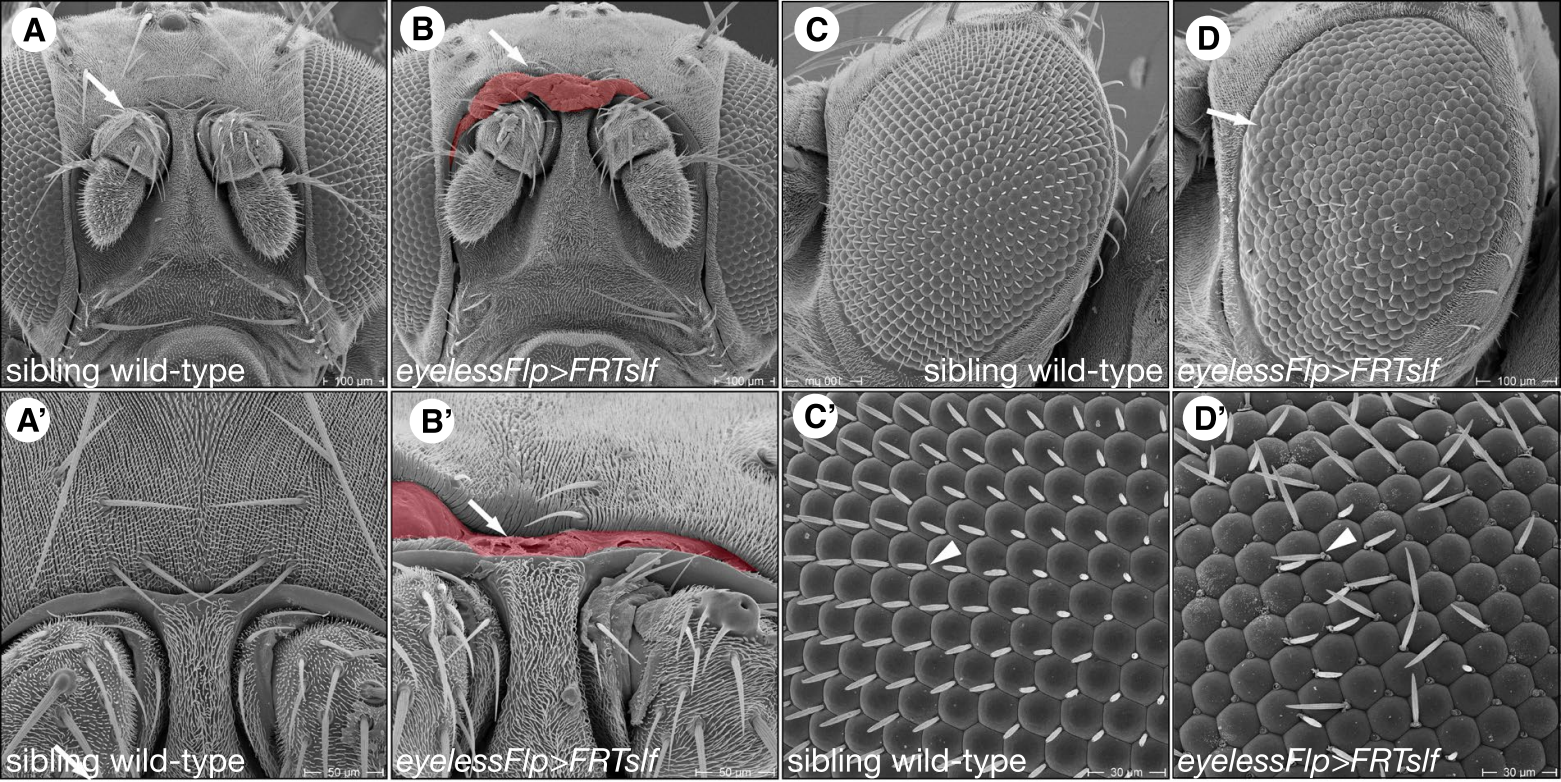
The ptilinum is disrupted in flies with down-regulated Slf. As shown in scanning electron micrographs, the head of the wild-type fly is composed of the large compound eye and sclerites bridged by rather narrow soft cuticular membranes that are not clearly exposed (**A,A’**). Homozygous *slf* mutant clones induced by the Flp/FRT system in the head of otherwise wild-type flies (*eylessFlp* > *FRT slf*) provoke disruption of especially the soft ptilinum at the forehead (arrow, **B,B’**) and the joining of the eye bristles with their basis (triangle, **D,D’**). Besides, the eye morphology is irregular. Occasionally, at the eye margin ommatidia are not clearly separated from each other (arrow in **D**). Possibly, eye shape defects and ommatidial disorganisation may be a consequence of deformed eye bristles.

In *slf* mutant larvae, the soft body cuticle is disorganised, the head skeleton, by contrast, that consists of a melanised and hard cuticle is unaffected (Fig. 1). This observation suggests that Slf is needed especially in soft but not hard cuticle. To test this assumption, using the Flp/FRT technique (see Materials & Methods), we generated *slf* mutant clones in adult heads that are composed of hard sclerites connected by soft joints. Flies harbouring *slf* mutant tissue in the head fail to eclose and die within the pupal case. The overall anatomy of their head appears to be normal (Fig. 5). However, the ptilinum, a soft and elastic cuticle that expands to break open the pupa case, is ruptured. We thus reckon that soft cuticle integrity requires Slf function. To corroborate this interpretation, we down-regulated *slf* activity in the whole body of developing larvae and pupa by RNAi (Supplementary Fig. S7). We observed that the cuticle in the leg joints, wing hinges, ventral abdomen and ptillinum of escaper flies were necrotic. The body parts with the hard cuticle appeared to be unaffected. These flies died in the pupal case or shortly after eclosion. In summary, our genetic experiments suggest that Slf is especially required in the unsclerotised, soft cuticle of larvae and adult animals.

### Slf cooperates with heme synthesis pathway in dityrosine layer formation

**Figure 6:**
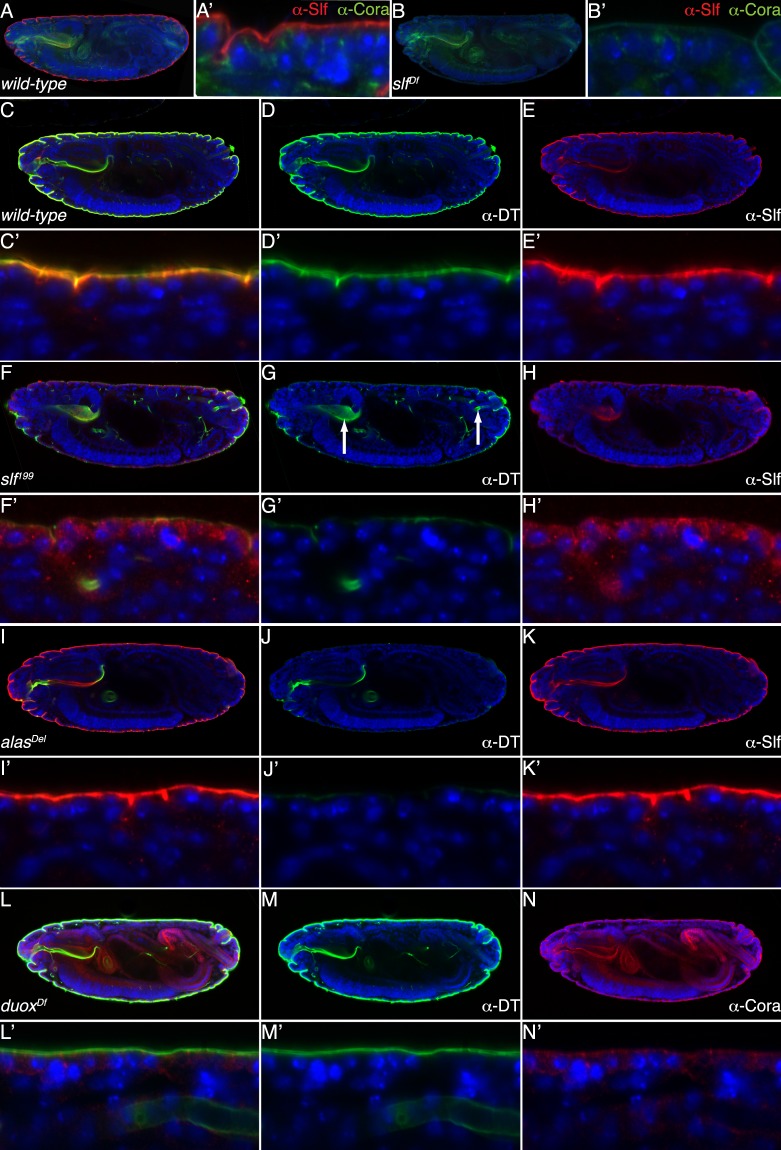
α-Slf antibody signal occurs in the cuticle of the embryos at late developmental stages. Probed with an α-Slf antibody (red), Slf is detected in the entire epidermal cuticle and the pharynx of *wild type* embryos at early stage 17 (**A,A’**). The head skeleton and the tracheal system are devoid of a Slf-positive signal. Embryos with a deleted *slf* gene do not show any α-Slf-signal (**B,B’**). Lateral boundaries between the cells are visualized by antibody staining against the junction protein Coracle (green) and the nuclei are visualized by blue DAPI staining (**A–B’**). The α-Slf antibody signal (red) co-localizes with the α-dityrosine antibody signal (α-DT, green) in the epidermal cuticle and the cuticle of the mouth hooks of the *wild-type* early stage 17 embryos (**C–E’**). In homozygous *slf*^*2L199*^ mutant embryos at the early stage 17 the α-Slf signal (red) occurs inside the epidermal cells, whilst the α-DT signal (green) is strongly reduced in the epidermal cuticle, contrary to the pharynx and the tracheae (arrows in **G**), where it remains strong (**F–H’**). In early stage 17 embryos homozygous for the mutation in the *alas* gene the α-Slf signal (red) occurs in the epidermal cuticle and the pharynx, whilst the α-DT signal (green) is strongly reduced in the whole body (**I–K’**). In early stage 17 embryos with a deletion of the *dual oxidase* (*duox*) gene, the α-DT signal (green) is unchanged compared to *wild-type* embryos (**L–N’**). The lateral boundaries between the cells are visualized by α-Coracle staining (red, **L–N’**).

Defects provoked by mutations in *slf* are reminiscent of those caused by mutation in *alas*, a gene encoding the delta-aminolevulinate synthase, which initiates the synthesis of heme (Fig. 1 & Supplementary Fig. S1)^22^. Is there a genetic and molecular relationship between Slf and heme synthesis pathway? In order to answer this question, we performed a series of genetic and histological experiments. First, we examined embryos double-mutant for *alas* and *slf* mutations. The phenotype of these embryos was comparable to the ones provoked by mutations in either of the genes (Supplementary Fig. S1). Assuming that both mutations represent loss-of-function situations, this observation suggests that these genes act in a common pathway. Consistently, reduction of larval *alas* or *slf* expression by RNAi caused a similar lethal phenotype (Supplementary Fig. S8). Second, we tested whether Slf localisation may depend on Alas function. Using our anti-Slf specific antiserum, we find that Slf localises to the cuticle of *alas* mutant embryos (Fig. 6). However, the thin L1 cuticle does not allow a more detailed localisation.

**Figure 7:**
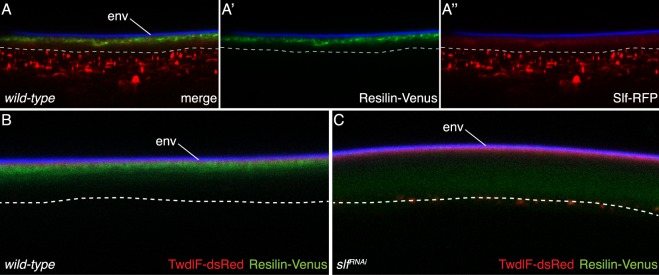
Resilin localization depends on Slf activity. In the cuticle of the third instar *Drosophila* larvae, the signal of RFP-conjugated Slf protein (**A–A”**, red) overlaps with the signal of Venus-conjugated Resilin (**A–A”**, green). The envelope emits a blue signal upon excitation by light with a wavelength of 405 nm. While in *wild-type* control third instar larvae the Resilin-Venus signal is confined to a narrow region in the upper cuticle part (**B**), it occurs in the whole procuticle of respective larvae with systemic down-regulated *slf* expression (**C**, green). The localization of the TweedleF-dsRed protein is unchanged in *slf* RNAi third instar larvae (**C**, red) in comparison to the *wild-type* control larvae (**B**). The dashed line marks the apical plasma membrane of epidermal cells.

The phenotype of *alas* mutant larvae has been linked with the breakdown of the dityrosine barrier^22^. Using a DT specific antibody (α-DT), we tested whether the dityrosine network may depend on the presence of Slf. We observed that dityrosine signal intensity is reduced in these animals in the integument cuticle, but not in the tracheal cuticle (not shown). This suggests that Slf might be either involved in dityrosine network formation or needed for the localisation i.e. stabilisation of dityrosinylated proteins to form a network. A well-known substrate protein modified by dityrosine links is Resilin^35^. We generated a Venus-tagged version of Resilin and co-expressed it with Slf-RFP in the cuticle of L3 instar larvae (Fig. 7). These chimeric proteins co-localise at the apical domain of Slf. Hence, Slf seems to be associated with dityrosinylated proteins. To further elucidate the relationship between Slf and Resilin, we expressed Resilin-Venus in third larvae with systemically RNAi-induced reduced *slf* expression. We observed that Resilin-Venus is mislocalised in these larvae (Fig. 7). This suggests that Slf might be responsible for either the delivery or the stabilisation of dityrosine-forming proteins to the correct position in the cuticle.

A well-known peroxidase involved in dityrosine formation in insects including fruit flies is the membrane-inserted Dual Oxidase Duox^36,37^. Using a-DT specific antibody, we tested the presence of dityrosines in homozygous mutant embryos deficient for *duox*. In these animals, the dityrosine signal was comparable to the signal in wild type embryos (Fig. 6). This suggests that either Duox is not involved in the formation of a larval dityrosine network, activity of maternally provided Duox is enough to catalyse the formation of the cuticle dityrosine network or another peroxidase may compensate decreased Duox activity.

Taken together, we conclude that Slf is a part of the dityrosine layer in the cuticle and the localisation of the dityrosinylated proteins to this layer depends on Slf activity.

## Discussion

The insect cuticle is a water resistant barrier withstanding the internal hydrostatic pressure and preventing uncontrolled transpiration and water penetration. Previously, we had shown that a heme-dependent pathway is required to generate a dityrosine-based waterproof matrix within the cuticle of the *D. melanogaster* larva^22^. Recently, we reported on the role of the ABC transporter Snustorr (Snu) and the extracellular protein Snustorr-snarlik (Snsl) in the construction of an envelope-based anti-penetration and anit-transpiration barrier in *D. melanogaster*^38^. In the present work, we propose that the C-type lectin Slf cooperates with the heme-biosynthesis pathway to stabilise the distribution of the cuticle dityrosinylated proteins, exemplified by Resilin. The network of dityrosinylated proteins, in turn, is needed for correct contact between chitin laminae within the procuticle and between the procuticle and the epicuticle.

### Slf is a cuticular C-type lectin

Analyses of the Slf protein sequence suggest that it is a putative secreted galactose-binding C-type lectin. In *D. melanogaster*, galactose residues are found on side branches of N-glycans and on a tetrasaccharide that links glycosaminoglycans (GAGs) to serine residues of certain membrane-bound proteins such as glypicans and syndecans^39^. Cuticle proteins have not been reported yet to harbour sugar moieties. Moreover, Slf is detected within the procuticle in *D. melanogaster* stage 17 embryos, especially accumulating at a distinct sheet at the apical border of the procuticle between the two zones marked by the cuticle proteins TwdlD and CPR67b. Based on these data, we assume that Slf is a cuticular C-type lectin contributing to late cuticle differentiation. Presumably, Slf exerts its function by binding an extracellular protein that carries a galactose. In principle, this finding is in line with data demonstrating that Slf (Clect27) is a cuticle protein that is essential for survival and needed for wing formation^21^. Moreover, it was shown that Slf is a substrate of the cross-linking enzyme transglutaminase that mediates covalent glutamine-lysine bonds. Down-regulation of transglutaminase expression, however, causes a mild cuticle phenotype compared to the strong *slf* mutant phenotype. Thus, taken together, Slf is a component of a composite extracellular network including non-essential covalent (glutamine-lysine bridges) and essential non-covalent (maybe galactose binding) interactions. This function is confined to the epidermal cuticle Slf being absent from the head skeleton and the tracheal system.

We find that Slf is present in other insects. Thus, the role of Slf in the soft cuticle of other insects is probably conserved. According to information from the beetle base on the putative orthologue of Slf in the red flour beetle *Tribolium castaneum* (http://ibeetle-base.uni-goettingen.de/details/TC013911), injection of double-stranded RNA into larvae is 100% lethal. A phenotype has not been reported. However, this result underlines that Slf is also essential in other insects than *D. melanogaster*.

### Slf function is independent of the envelope

Classically, the outermost cuticle layer called envelope has been considered to be the bona fide desiccation barrier. In a recent work, we demonstrated that the extracellular protein Snsl and the ABC transporter Snu contribute to the establishment of the envelope in turn ensuring desiccation as well as penetration resistance^38^. The function of Snu is obviously conserved in other insects^40,41^. The envelope of *slf* mutant larvae is normal at the ultrastructural level. In addition, cuticle impermeability to xenobiotics is maintained in these larvae indicating that Slf is dispensable for an inward barrier. Furthermore, the procuticle is not disrupted in *snu* or *snsl* mutant larvae. Based on these evidences, we conclude that Slf and Snu/Snsl act in different pathways or mechanisms designed to establish a cuticular barrier preventing especially water loss.

### Slf is required especially in the soft unsclerotised cuticle

Elimination or reduction of Slf function especially affects the integrity of soft cuticle types including the larval body cuticle, the joint cuticle and the ptilinium. By contrast, hard cuticle types are largely unaffected. The major difference between hard and soft cuticles is the presence of an elaborate exocuticle in the hard cuticle that, as at the upper portion of the procuticle, consists of a sclerotised chitin-protein matrix. Based on this histological difference, we hypothesise that the region between the unsclerotised procuticle - called endocuticle in the hard cuticle - and the epicuticle is a region where components are cross-linked either by catecholamines (sclerotized exocuticle) or by dityrosines (soft cuticle). This region is apparently needed to prevent massive water loss through the cuticle.

### Slf is involved in organising cuticle compactness through production or stabilisation of the dityrosine network

Mutations in *slf* are embryonic lethal. Loss of Slf function entails massive water loss. By fluorescence microscopy, we show that the outer TwdlD-layer of the cuticle detaches from the inner CPR67b-layer of the cuticle in respective ready-to-hatch larvae. In addition, by transmission electron microscopy, we show that the procuticle of these larvae is loose. Thus, Slf is needed for compactness in the procuticle as well as the attachment of the TwdlD- to the CPR67b-layer within the cuticle.

**Figure 8:**
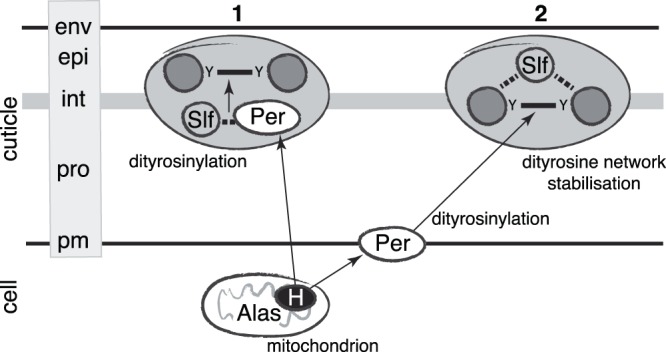
Slf promotes dityrosine formation or stabilises the diytrosine network. Our data allow proposing two alternative scenarios of Slf function. Either Slf assists directly a haem-dependent peroxidase (Per) at dityrosinylation of cuticle proteins such as Resilin (1), or it is needed to localize and stabilize the dityrosine network (solid lines) in the interface (int) between the epi- (epi) and procuticle (pro). Stabilization of the interface or interaction with a peroxidase may require sugar binding (dotted lines). The peroxidase may be inserted into the plasma membrane (pm) or extracellular; to simplify the scheme we have indicated only one possibility for each alternative scenario. Haem (H) is produced in the cytoplasm involving mitochondrial Alas. env envelope.

The detachment of parts of the larval cuticle from the body is reminiscent of the *alas* mutant phenotype^22^. This suggests that Slf and Alas may contribute to the same structure in the cuticle. Alas is involved in the production of heme that is a co-factor of a yet unidentified oxidase catalysing the formation of a dityrosine network within the cuticle at the end of embryogenesis. We find that the cuticular dityrosine signal is reduced in *slf* mutant embryos and that the dityrosinylated cuticle protein Resilin is mislocalised in these animals. We conclude that Slf is required either for production or stabilisation of the dityrosine network that constitutes a barrier against water loss (Fig. 8).

Similarly, in vertebrates, galectin-3 forms an impermeable 500 nm thick lattice through the interaction with mucins at the surface of the ocular epithelium^42^. The presumed association of Slf with galactose-residues in a group of N-glycans or GAGs, its incorporation in a dityrosine and Gln-Lys network would in an analogous manner stabilise extracellular proteins required for cuticle integrity and barrier function. Slf is, hence, an adapter-like protein that glues different cuticular networks. Overall, we suspect that lectins may play a key role in ECM organisation.

## Materials and Methods

### Standard fly work and microscopy

Mutations and deficiencies (Table 1) were kept over balancers harbouring GFP or YFP constructs expressed under the control of either *Krüppel* or *Deformed*. This allows identification of homozygous mutant embryos, which lack any GFP/YFP expression. They were collected on apple juice agar plates garnished with a spot of yeast paste. For dextran injection experiments, embryos (n > 20) were dechorionated with bleach, and those of the desired stage were selected by hand, and dried on silica granulate for 4 minutes. 3kD dextran coupled to rhodamine (Thermo-Fisher) were dissolved at a 10 mg/ml concentration in Sörensen injection buffer. For injections, these solutions were mixed at a 1:1 ratio, resulting in a 5 mg/ml concentration for each labelled dextran. Starting immediately after injection, behaviour of the fluorescence signal was monitored for about one hour using a Zeiss Axiophot microscope.

**Table 1:** Fly stocks used in this work.

Line	Description	Origin
*slf* ^*IJ83*^	Point mutation in *slf*	Ref.^53^
*slf* ^*2L–199*^	Point mutation in *slf*	Ref.^49^
Df(2 L)ED250	Deficiency uncovering *slf*	BDSC
Df(2L)BSC225	Deficiency uncovering *slf*	BDRC
CHS322-140E11	Insertion including *slf* and *CG3294*	Pacman Resources
3244R-3^II^	*slf* RNAi	NIG, Japan
P{KK101189}VIE-260B	*alas* RNAi	VDRC, Austria
#46945	*CG4115* RNAi	VDRC, Austria
#30742	*CG6055* RNAi	VDRC, Austria
TwdlD-dsRed	Cuticle marker	Ref.^27^
TwdlM > Cpr67B-RFP	Cuticle marker	Own stock
TwdlM > Slf-RFP	Cuticle marker	Own stock
UAS > Res-Venus	Cuticle marker	Own stock
Tb-GFP	Cuticle marker	Own stock
ObstE-GFP	Cuticle marker	Ref.^34^
*alas*^*DeltaKG10015*^	Deletion in *alas*	Ref.^22^
*alas*^*DeltaKG10015*^*; slfIJ83*	Double *alas-slf* mutant	Own stock
*DuoxK*	Dual oxidase point mutation	BDSC
*da*-Gal4	Ubiquitous Gal4	BDRC
*knk*-Gal4	Epidermal Gal4	Own stock
*btl*-Gal4	Tracheal Gal4	BDRC
FRT2L	Flipase Recognition Target sequence	BDRC
*eye* > *Flp*	Flipase in the head	BDRC
*wild-type*	Wild-type Oregon	BDSC

For cuticle preparations, larvae (n > 50) were deposited on a glass slide in Hoyer’s medium^43^ covered by a coverslip and incubated at 65 °C or 80 °C overnight. They were examined by Nomarski microscopy on a Zeiss Axiophot microscope. Cuticle auto-fluorescence (envelope) was examined after excitation with a UV light source or a 405 nm laser (see below). For immunofluorescence microscopy, dechorionated embryos (n > 100) were fixed in Hepes buffered 3,7% formaldehyde for 20 minutes at room temperature, devitellinized and incubated with the respective antibodies, which were detected with appropriate secondary antibodies. Stained embryos were viewed on a Zeiss Axiophot, Zeiss LSM 710 or 880 confocal microscopes. For excitation and detection of fluorophores or fluorescent proteins, the following laser/filter combinations were used: 405 nm/BP 409–499 nm (envelope auto-fluorescence, DAPI), 488 nm/BP 493–588 nm (ObstE-GFP, Alexa-488 antibody), 514 nm/BP 519–588 nm (Resilin-Venus), 561 nm/BP568-712 (Alexa-561 antibody, Slf-RFP, CPR67b-RFP, TwdlD-dsRed).

For permeability experiments, embryos (n > 20) were dechorionated, devitellinized and incubated in bromophenol blue solution following the protocol described in^30^. For electron microscopy, specimens were prepared following the protocol described in Moussian and Schwarz^44^. Samples for scanning electron microscopy (SEM, n = 10) were prepared and analysed as published recently^45^. For live imaging, ready-to-hatch larvae carrying fluorescent cuticle markers were put on a glass slide into a drop of Halocarbon oil 700 (Sigma) and covered with a coverslip (n > 20). Cuticle detachment was monitored using a Leica DMI8 fluorescent microscope. For live imaging of second and third instar larvae, larvae were anesthetised with ether, mounted in halocarbon oil on a glass slide and covered with a coverslip (n > 20). Fluorescence was observed on a Zeiss LSM 880 microscope. Images were prepared using Adobe Photoshop and Illustrator CS6 software.

### Generation of transgenic flies

For the generation of flies carrying the transposon with CPR67B-RFP, 500 bps of the *twdlM* promoter, the coding region of *cpr67B* the and *rfp* coding region^27^ were cloned into the pW8 vector to create the transposon pW8*twdlMP*:*cpr67B*:*rfp*, which was injected into *w*^1118^ embryos. The *twdlM* promoter and the *cpr67B* gene were used for this study because according to the modEncode database^46^, they are especially active in the last hours of embryogenesis when the *slf* mutant phenotype becomes manifest.

The Pacman CH322-140E11 that harbours an *attB* site^47^ was injected into flies with the *attP* landing site PBac{yellow[+]-attP-9A}VK00030 at 50E1 on the right arm of the second chromosome. This work was carried out by the BestGene company (Chino Hills, CA, USA). This insertion was recombined to the chromosome with the *slf* ^*2L–199*^ allele (left arm of the second chromosome).

The *knk*-Gal4 transposon was generated by cloning 500 bp of the *knk* 1^st^ intron sequence that is active in the epidermis^48^ upstream of the *gal4* sequence in the pGaTB vector. This transposon was introduced into the genome of *w*^1118^ flies by standard transgenesis.

### Generation of homozygous slf mutant clones

The *slf* ^*2L199*^ allele was induced on a chromosome carrying FRT (Flipase Recognition Target) sequence^49^. These flies were crossed to flies carrying a lethal mutation on a 2nd chromosome with FRT sequence and expressing Flipase in the head driven by the *eyeless* promoter (*eye* > *flipase*). The progeny carried *slf*, FRT on one chromosome, FRT, ubi-GFP on the homologous chromosome and expressed Flipase in the head of developing flies. As a consequence of the Flipase activity, *slf* homozygous clones were generated in the head of developing pupae.

### RNA interference

To generate flies expressing hairpin RNA against *slf* (*slf* ^*RNAi*^) in the epidermis of pupae the UAS/Gal4 system was used^50^. Flies carrying *slf* ^*RNAi*^ under the control of the UAS promoter (*UAS* > *RNAi-slf*, accession number NM_135014.2 from NIG-Fly, Kyoto, Japan) were crossed with flies harbouring *Gal4* under the control of the promoter of the *knickkopf* gene (*knk-*Gal4). For a systemic expression of UAS-constructs a combination of *da*-Gal4 and 7063-Gal4 (both Bloomington Stock Center) named as L370-Gal4 was used.

### Molecular Biology and sequence analyses

Standard molecular techniques (PCR, sequencing) were applied to identify and characterise the *slf* gene as presented in Fig. 3. Sequences were analysed using the BLAST software at flybase (flybase.org) and NCBI (https://blast.ncbi.nlm.nih.gov). Protein domains were identified with the Conserved Domains option at NCBI. For multiple sequence alignment, the Clustal Omega software at EMBL-EBI (https://www.ebi.ac.uk/Tools/msa/clustalo/) was used. Structural alignments as shown in Supplementary Fig. 5 were performed using the online HHpred software (https://toolkit.tuebingen.mpg.de/#/tools/hhpred).

For *in situ* hybridization experiments, we followed the protocol described in^51^. In brief, the *slf* CDS was cloned into the pCR2.1 vector (ThermoFisher). Sense and anti-sense probes were produced from independent inserts with opposing direction using BamHI linearized vectors to transcribe a Digoxigenin-labelled probe via the T7 promoter (T7 RNA polymerase, Roche, Germany). The probes were detected by an anti-DIG antibody conjugated with Alkaline Phosphatase (Roche) followed by staining with NBT/BCIP (Roche). More than 40 embryos were analysed for each probe.

### Production of a Slf-specific antibody

In principle, we followed a standard protocol to generate a polyclonal Slf specific antibody^52^. In brief, for antibody production via rabbit immunisation, a GST-tagged version of the Slf protein excluding the N-terminal signal peptide was produced in bacteria using the expression vector pGEX-4T1 (GE Healthcare). After purification of the recombinant protein on Glutathion Sepharose 4B beads (GE Healthcare), it was diluted in complete Freund’s adjuvant (CFA, 100 µg protein in 500 µl CFA) and injected into a rabbit at the Max-Planck Institute for Developmental Biology in Tübingen, Germany. Antiserum production was boosted every other week and blood was collected in between boosts for 12 weeks. After centrifugation of the blood samples, antisera were probed on Western Blots and on embryos in immune-detection experiments; the optimal dilution in both experiments is 1:500.

## Supplementary information

Supplementary figures
