# Supplementary material for: The putative C-type lectin Schlaff ensures epidermal barrier compactness in Drosophila
Source: Sci Rep. 2019 Mar 29;9:5374. doi: 10.1038/s41598-019-41734-9 (PMC6440989; doi:10.1038/s41598-019-41734-9)
Supplement: Supplementary file 1 — Supplementary figures [file 41598_2019_41734_MOESM1_ESM.pdf]

The putative C-type lectin Schlaff ensures epidermal barrier compactness in *Drosophila*

Renata Zuber[1,2], Khaleelulla Saheb Shaik[2], Frauke Meyer[2], Hsin-Nin Ho[2], Anna Speidel[2], Nicole Gehring[2], Slawomir Bartoszewski[3], Heinz Schwarz[4] & Bernard Moussian[5]

1 Applied Zoology, Technical University of Dresden, Zellescher Weg 20b, 01217 Dresden, Germany.

2 University of Tübingen, Interfaculty Institute of Cell Biology, Section Animal Genetics, Auf der Morgenstelle 15, 72076 Tübingen, Germany

3 Rzeszow University, Department of Biochemistry and Cell Biology, ul. Zelwerowicza 4, 35-601 Rzeszów, Poland

4 Max-Planck-Institut für Entwicklungsbiologie, Microscopy Unit, Spemannstr. 35, 72076 Tübingen

5 Université Côte d’Azur, CNRS, Inserm, Institute of Biology Valrose, Parc Valrose, 06108 Nice CEDEX 2, France

Supplementary figures

Fig. S1 Dityrosine distribution depends on Slf but not on Duox

The *wild-type* ready-to-hatch living larva fills the entire egg (A). Ready-to-hatch larvae with eliminated or reduced *slf* function (slf^{IJ83}, slf^{2L-199}, *slf deficiency*, slf^{RNAi}) are contracted and the space between the embryo and the egg case is filled with liquid (B-E). As in control animals, their tracheal system is air-filled (white triangles) and the head skeleton seems to be unaffected. The phenotype of the homozygous mutants carrying loss-of-function insertion in the *alas* (F) gene is reminiscent of the *slf* phenotype, but, additionally the tracheae are not air-filled. The phenotype of the *slf alas* double mutant larva resembles *alas* mutant embryos (G). The tracheae of the homozygous mutants in the *dual oxidase* (*duox*) gene are not air-filled, but the larvae do not contract in the egg (H).

After freeing from the egg and keeping the living larvae in halocarbon oil under the coverslip, the *wild type* larvae larvae stretch and their cuticle lines the body surface (I), whilst the cuticle of the *slf* mutant (J,K), *alas* mutant (L) and the *slf, alas* double mutant (M) larvae to a lesser or greater extent detaches from the body surface. In

duox mutant larvae only a thin layer of the cuticle, probably the envelope detaches from the surface (N).

In Hoyer's cuticle preparations, the envelope as visualized by a 405nm laser (blue) lines the body surface of the wild-type larva (O). In *slf* (P, Q) and *alas* (R) mutant larvae, the envelope forms small blisters at the ventral (vs) and large blisters at the dorsal side (ds) of the body. In *slf, alas* double mutant larvae, it detaches from the whole body forming large blisters (S). In *duox* mutants it forms small blisters on the ventral side of the body only (T).

Fig. S2 Cpr67B-RFP and TwdlD-dsRed localise to distinct regions of the cuticle

As shown in L2 larvae, TwdlD-dsRed (red signal) localises close to the surface auto-fluorescence (blue signal) exited by 405nm. As shown in L3 larvae, Cpr67B-RFP (red signal) localises in a broad region underneath the auto-fluorescing layer (blue signal). The epidermal cells are marked by a cytoplasmic GFP protein (green).

Fig. S3 Slf is not needed for inward barrier function

The cuticle of the living *wild-type*, *slf*, *alas* and *duox* homozygous mutant ready-to-hatch larvae is impermeable for bromophenol blue (bpb). The upper panel shows the larvae before incubation and the lower panel after incubation with bpb. Larvae homozygous mutant for *snsl* exhibiting a defective envelope are permeable for bpb, which leaks into the larvae and stains them with a dark blue colour.

Fig. S4 Septate juctions of the slf mutant larvae are normal

Septate junctions (SJ) connect neighbouring epidermal cells as shown in electron micrographs of late *wild type* embryos (A). Comparably, septate junctions of the *slf* mutant larvae are unchanged (B). In larvae carrying mutation in gene encoding septate junction component Coracle (*cora*), the septate junctions are not present (C). cut = cuticle.

Epidermal cells (asterisk) of stage 16 *wild type* (D) or *slf* mutant embryos (E) contain particles (arrow) of 10 kDa dye-conjugated dextran that was injected in their haemolymph. By contrast, in stage 16 *coracle* (*cora*) mutant embryos (F), the dextran signal is also detected in lines probably representing the lateral membrane (triangle).

Fig. S5 Full version of the Western blot shown in Fig. 3

The antiserum raised against CG3244 recognises a protein with a molecular mass around 27kDa in extracts of wild-type larvae that is missing in *slf*^{dJ83} mutant larvae.

Fig. S6 The Slf protein is a C-type lectin

Using the HHPred software (https://toolkit.tuebingen.mpg.de/#/tools/hhpred), we detected mammalian L-Selectins (here human) as a distant structural homolog of Slf in the SCOP database (A). Especially Cysteine (C) and Tryptophan (W) residues (*) that are important for the correct folding of the protein, are conserved. The amino acids mutated in Slf-versions are highlighted in red. The QPD sugar-binding signature is boxed. The EPN sugar-binding domain in L-Selectin is underlined. In total, *D. melanogaster* has two additional C-type lectins that are homologous to Slf (signal peptide light grey box, C-type lectin domain dark grey box), namely CG4115 and CG6055 (B). Along with extensive stretches of sequence identity, the QPD signature (mid-grey box) and the amino acids G and E in mutated Slf-versions (tone-less boxes) are conserved.

Fig. S7 Flies with down-regulated Slf activity show soft cuticle damages and necrosis

Expression of *slf* was reduced by RNAi (slf^{RNAi}) using the epidermal Gal4 *knk*-Gal4. Most of *knk*-Gal4, slf^{RNAi} animals die before eclosion. However, a few flies of this genotype do eclose. By light microscopy, in these escaper flies, we observe necrosis in the soft cuticle regions like the forehead (arrow, B), the dorsal abdominal region (asterisk, D), at joints (arrow, F) and the wing hinge (arrowhead, H) in comparison to the intact cuticle in *wild-type* flies (A, C, E and G).

Fig. S8 Down-regulation of slf or alas causes a similar larval phenotype

The wild-type L1 and L3 larvae have a slender body shape (A,D). L3 and L1 larvae with down-regulated *slf* expression in the epidermis and tracheae or down-regulated ubiquitous *alas* expression induced by RNAi (slf^{RNAi}; *knk*-Gal4 and $alas^{RNAi}$; *L370*-Gal4, respectively) are podgy and have melanised injuries on their surface (arrows, B,C).

We were unable to compare the same larval stages for down-regulation of *slf* or *alas*, because slf^{RNAi}; *L370*-Gal4 larvae die within the egg case showing the *slf* mutant phenotype, and $alas^{RNAi}$; *knk*-Gal4 do not show any phenotype.

Figure S1

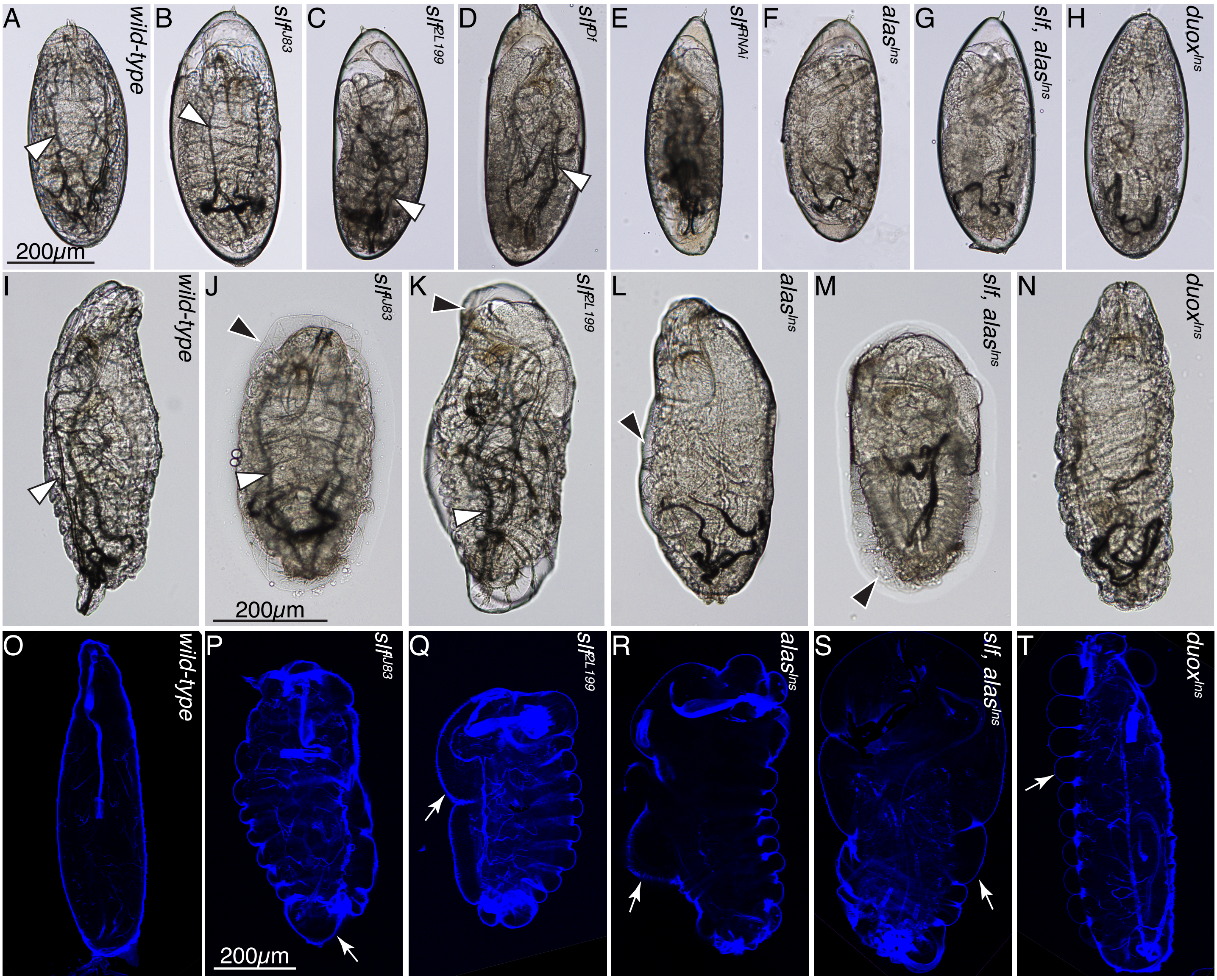
A wild-type
B slf
C slf
D slf
E slf
F alas
G slf, alas
H duox
200μm
I wild-type
J slf
K slf
L alas
M slf, alas
N duox
200μm
O wild-type
P slf
Q slf
R alas
S slf, alas
T duox
200μm

Figure S2

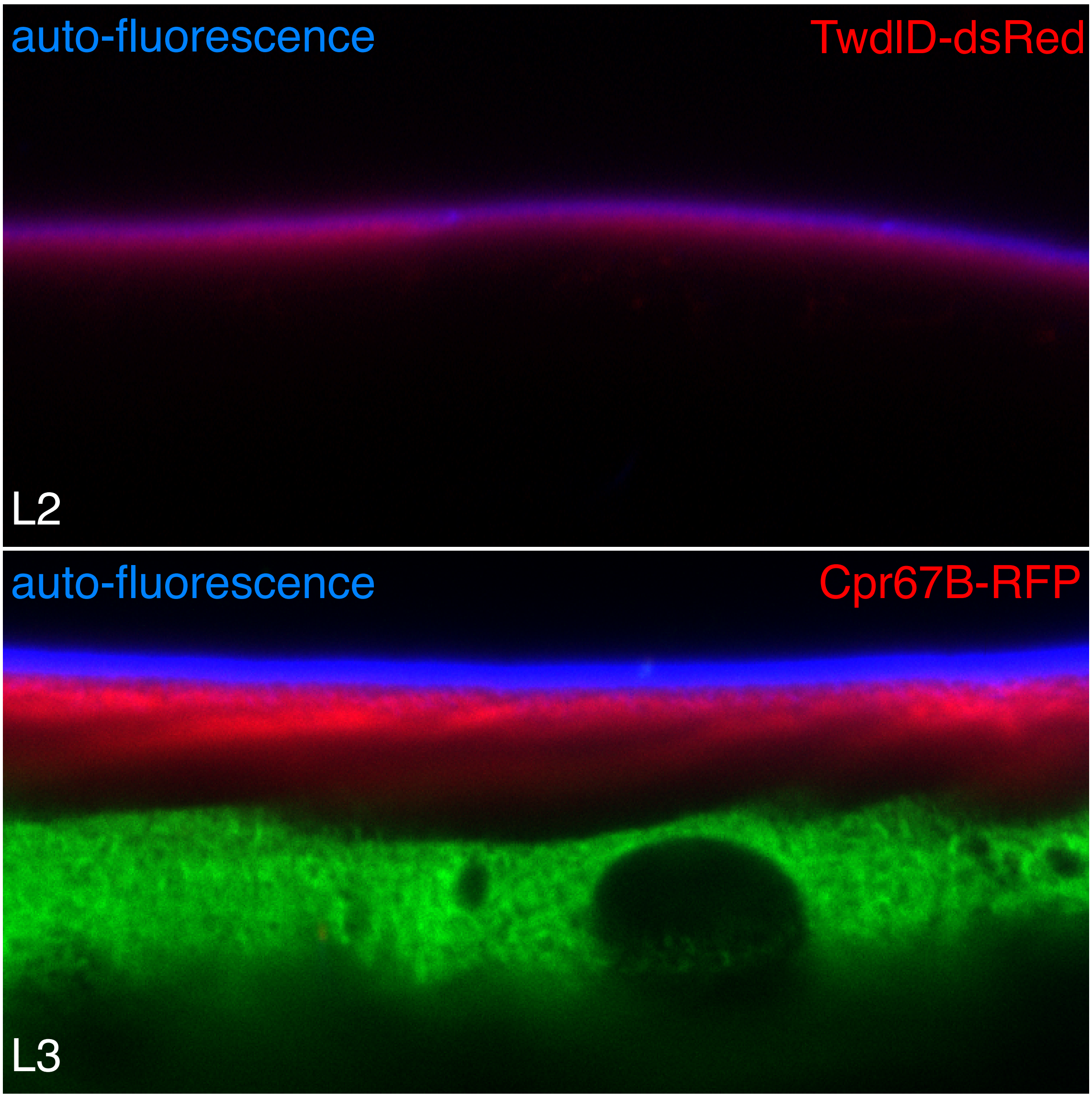

auto-fluorescence
TwdlD-dsRed
L2
auto-fluorescence
Cpr67B-RFP
L3

Figure S3

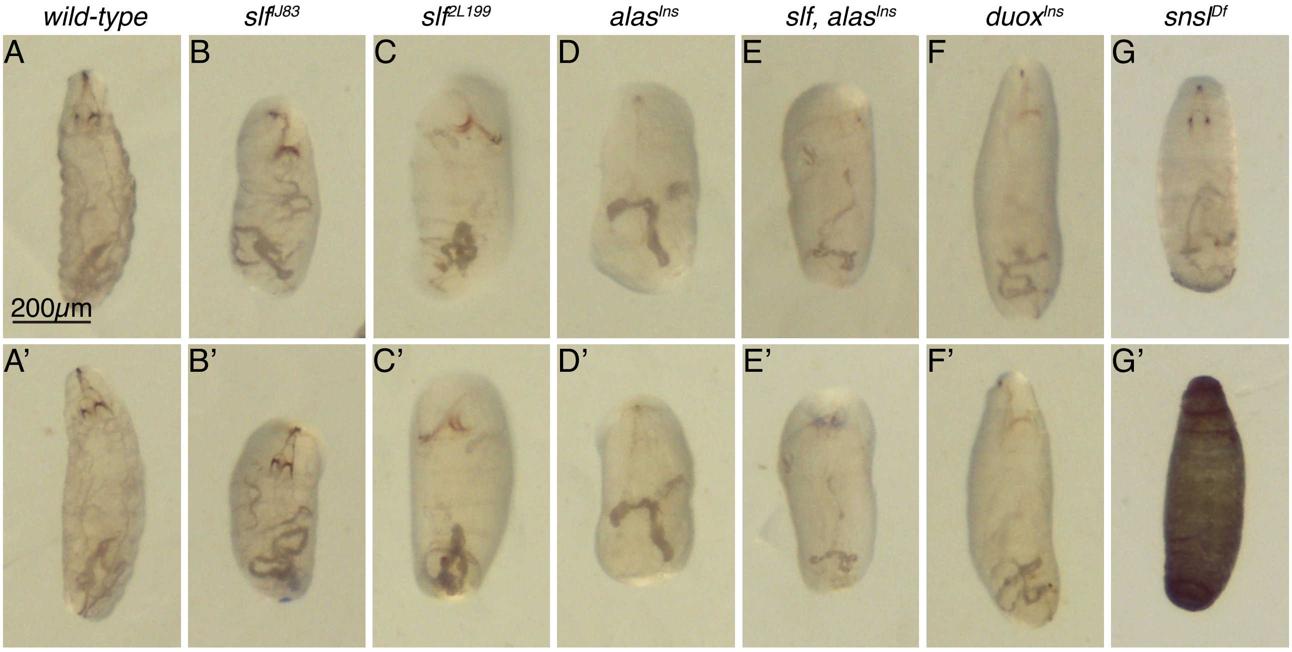

wild-type
slf1J83
slf2L199
alasIns
slf, alasIns
duoxIns
snslDf
A
B
C
D
E
F
G
200μm
A'
B'
C'
D'
E'
F'
G'

Figure S4

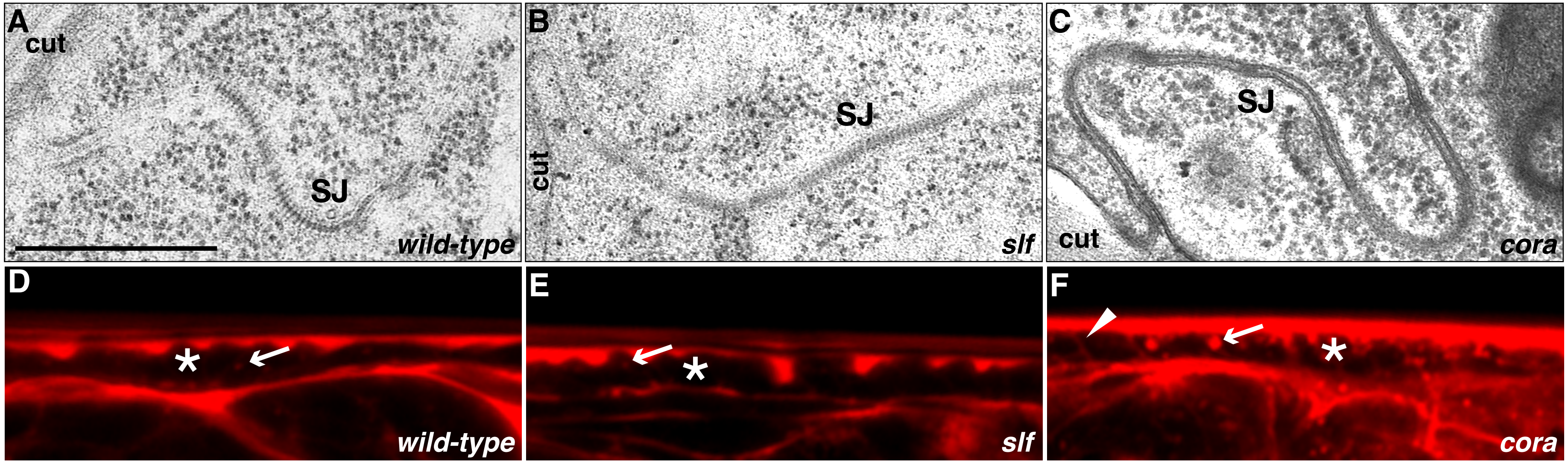

A
cut
SJ
wild-type
B
cut
SJ
slf
C
SJ
cut
cora
D
wild-type
E
slf
F
cora

Figure S5

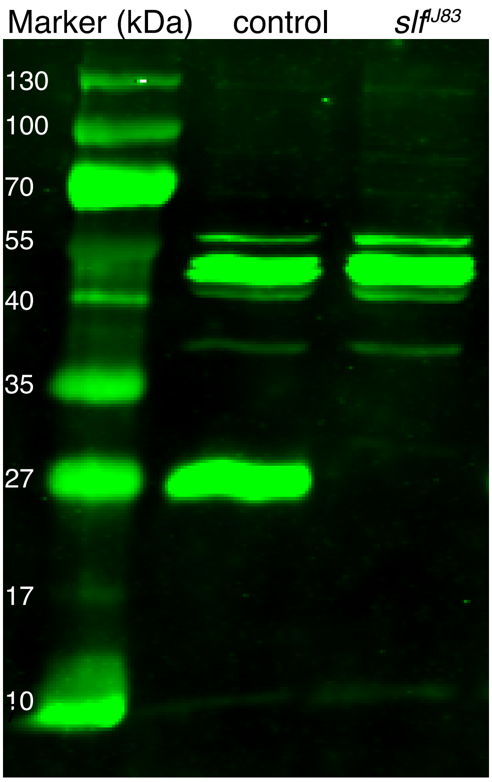

Figure S6

A

```
                          *                                         *
Slf          28    TKPGRFLSLPVPAKCASRPKE----FSYRGKNM----FLTTH-----VPALANKKVDWLD  74
                   T+ G   ++  P KC S  ++     F   G  M    FL  H         + K ++W
L-Selectin   6     TREGPSKAMIFPWKCQSTQRDLWNIFKLWGWTMLCCDFLAHHGTDCWTYHYSEKPMNWQR  65

                       *                            *
Slf          75    GRNLCREYCMDLVALETQEKNNLIFRVIQQNDVPYIWTAGRICDFAGCENRPDLEPKTVY  134
                    R   CR+    DLVA++ + +   + + +  +   Y W  G                K
L-Selectin   66    ARRFCRDNYTDLVAIQNKAEIEYLEKTLPFSR-SYYWI-GIR--------------KIGG  109

                    * *                          *                     *
Slf          135   GWFWSATREKIQATNRIPQGWGYNPWSQTGHKKRPQPDNAEYDINQTKEQCLSVLNNVYN  194
                    W W  T + +       + WG                + E +  + KE C+ +
L-Selectin   110   IWTWVGTNKSLTEE---AENWG----------------DGEPNNKKNKEDCVEIYIKRNK  150

                      *    *       *
Slf          195   DGIAWHDVACYHEKPVIC  212
                   D   W+D AC+  K  +C
L-Selectin   151   DAGKWNDDACHKLKAALC  168
```

B

```
CG6055         ------MSVLRALMFLAFGATVALAQRR-----LALPDPRSCANRVRHASYRDARGVSHS
CG4115         -MKAFIIAGVCVLSVLSLGS-AQFQNGR-----LEPPNPQLCAQRVIHEKTPDGKG----
Slf            MKVTLAIATFCVVMACSHAARTTTTTATKPGRFLSLPVPAKCASRPKEFSYR-GKN----
               :   : :: . .:    :  .: .              :*  *  *   **.*   . .    .:.

CG6055         YFFSWEHAPTRSLEVDWLDARNICRRHCMDAVSLETPQENDFVKQRIARGNVRYIWTSGR
CG4115         YFFSWRDPQLKGVEEDWLTARNYCRRRCMDSVSLETSLENEWIKQYVVRENVKYIWTSGR
Slf            MFLTTHVPALANKKVDWLDGRNLCREYCMDLVALETQEKNNLIFRVIQQNDVPYIWTAGR
                *::  .  .     .  :  ***  .**  **.  ***  *:***    :*:  :  :  :  :  :*  ****:**
                                                                          slf^IJ83

CG6055         KCNFAGCD-RPDLQPPNENGWFWSGSGAKIGPTSQRNTGD----WSSTGGYQQPQPDNRE
CG4115         LCDFKGCD-RPDLQPTNINGWFWTATLQKLAPTTERNQGD----WSPTGGIGLPQPDNRE
Slf            ICDFAGCENRPDLEPKTVYGWFWSATREKIQATNRIPQGWGYNPWSQTGHKKRPQPDNAE
                *:*  **:  ****:*  .   ****:.:   *:  .*..    *      ** **    ***** *

CG6055         AAQ-GNDESCLSILNNFYNDGIKWHDVACHHIKPFVCEDSDELLNFVRSRNPNVRL  219
CG4115         YKQNGAPENCLALLNQFYNDGVNWHDVACHHKKSFVCEENDALLKYVRYTNPNLRI  220
Slf            YDINQTKEQCLSVLNNVYNDGIAWHDVACYHEKPVICEDNEELLRYVAATNPGIRL  231
                         *.**::**:.****:  ******:*  *..:**:.:  **.:*    **.:*:
                      slf^2l-199
```

Figure S7

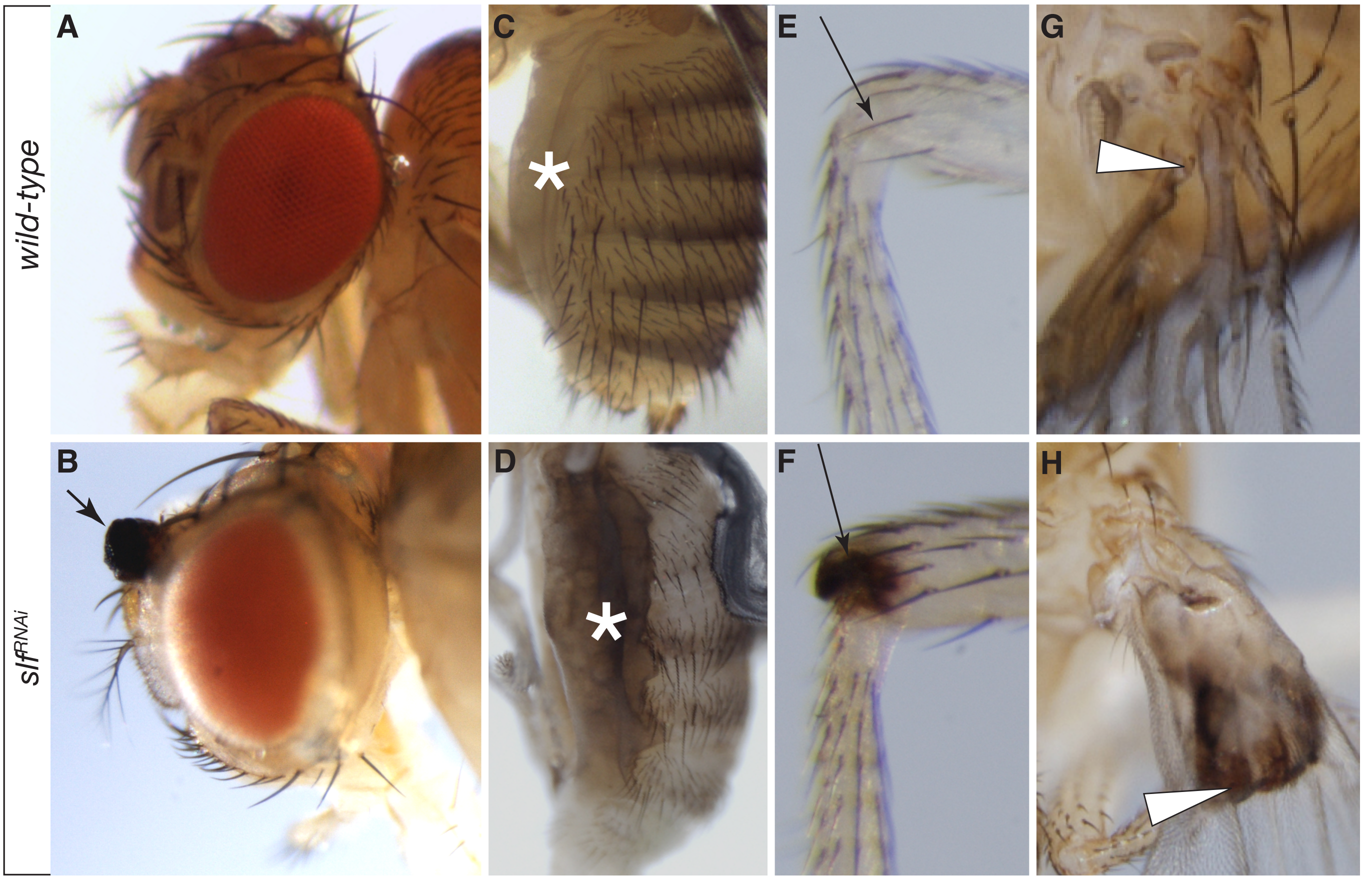

Figure S8

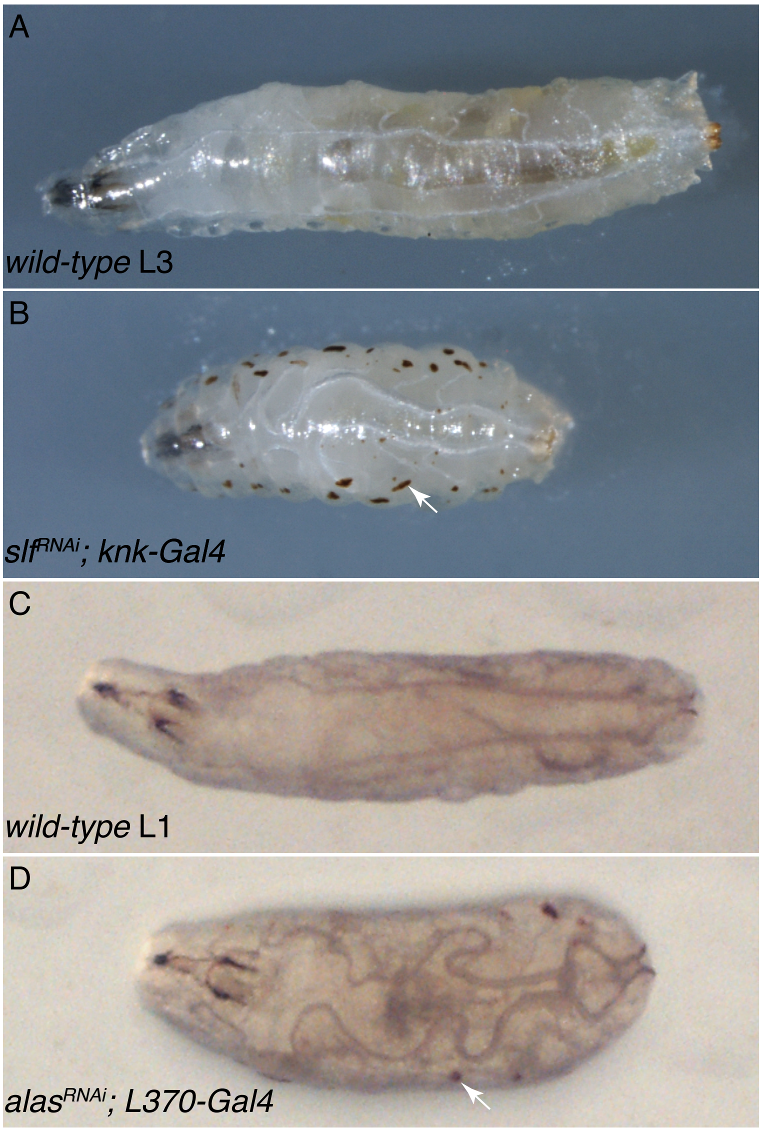
A
wild-type L3
B
slf^{RNAi}; knk-Gal4
C
wild-type L1
D
alas^{RNAi}; L370-Gal4
